# Complete Genome Sequence Obtained by Nanopore and Illumina Hybrid Assembly of Xanthomonas arboricola pv. juglandis CPBF 427, Isolated from Buds of a Walnut Tree

**DOI:** 10.1128/MRA.00085-21

**Published:** 2021-03-11

**Authors:** Miguel Teixeira, Camila Fernandes, Cátia Chaves, Joana Pinto, Fernando Tavares, Nuno A. Fonseca

**Affiliations:** a Centro de Investigação em Biodiversidade e Recursos Genéticos (CIBIO), InBIO-Laboratório Associado, Universidade do Porto, Vairão, Portugal; b Faculdade de Ciências (FCUP), Departamento de Biologia, Universidade do Porto, Porto, Portugal; c Instituto Nacional de Investigação Agrária e Veterinária (INIAV), Unidade Estratégica de Investigação e Serviços de Sistemas Agrários e Florestais e Sanidade Vegetal, Oeiras, Portugal; University of Rochester School of Medicine and Dentistry

## Abstract

We report the genome sequence of Xanthomonas arboricola pv. juglandis strain CPBF 427, which was isolated from early-season buds of a diseased walnut tree, suggesting overwinter potential. This study provides a consistent genomic reference for this pathovar and may contribute to addressing the overwinter survival of these walnut pathogens.

## Abstract (precis)

We report the genome sequence of Xanthomonas arboricola pv. *juglandis* strain CPBF 427, which was isolated from early-season buds of a diseased walnut tree, suggesting overwinter potential. This study provides a consistent genomic reference for this pathovar and may contribute to addressing the overwinter survival of these walnut pathogens.

## ANNOUNCEMENT

Xanthomonas arboricola pv. juglandis (*Gammaproteobacteria* class, *Xanthomonadales* order, *Xanthomonadaceae* family) is the etiological agent of walnut (Juglans regia L.) diseases affecting leaves, fruits, branches, and trunks (1–3). Bacteria belonging to this pathovar are characterized by considerable genomic diversity and are well spread through the main worldwide walnut-producing regions, causing important economic losses (4, 5).

This announcement reports the whole-genome sequence of X. arboricola pv. juglandis strain CPBF 427 (LMG 31039), which was isolated early in the spring of 2016 from dormant buds of a walnut tree (located in a public park in Loures, Portugal) that later in the season showed symptoms of walnut bacterial blight. These data show that strain CPBF 427 is present early in the growing season and likely is able to overwinter in the tree, thus probably favoring the infection of emerging leaves (6). X. arboricola pv. juglandis CPBF 427 was obtained from asymptomatic buds picked from the same branch. The isolation procedure started with bud excision, followed by superficial disinfection with 70% ethanol and sterile distilled water (SDW) and then maceration of buds with 5 ml of SDW in extraction bags (Bioreba AG, Reinach, Switzerland). For genome sequencing with Illumina and Oxford Nanopore Technologies (ONT) MinION platforms, strain CPBF 427 was grown on bacterial culture medium M2 (yeast extract, 2 g liter^−1^; Bacto peptone, 5 g liter^−1^; NaCl, 5 g liter^−1^; KH_2_PO_4_, 0.45 g liter^−1^; Na_2_HPO_4_·12H_2_O, 2.39 g liter^−1^) at 28°C and 100 rpm, followed by genomic DNA extraction using the E.Z.N.A. bacterial DNA purification kit (Omega Bio-tek, Norcross, GA), as described previously (7). Illumina sequencing was outsourced to GATC Biotech, AG (Konstanz, Germany), and conducted using standard library preparation and an Illumina HiSeq platform with 2 × 150-bp paired-end reads, resulting in 7,562,695 reads with 456× coverage (7, 8). For Nanopore sequencing, libraries were prepared with the SQK-LSK109 kit and multiplexed using the EXP-NBD104 barcoding kit. Sequencing was performed on a MinION sequencer, using a R9.4.1 flow cell. Reads were base called and demultiplexed using Guppy v3.4.1 (high-accuracy base-calling mode), which produced 14,302 reads with a mean length of 6,358 nucleotides (nt) (±7,722 nt), a maximum read length of 84,756 nt, and an *N*_50_ value of 13,332 nt. The reads were assembled *de novo* following a hybrid Nanopore-Illumina approach using Unicycler v0.4.8 (9) and annotated with PGAP v2020-07-09.build4716 (10). Assembly metrics were calculated using QUAST v5.0.2 (11). The assembly quality and completeness were assessed using BUSCO v4.0.6 (parameter: lineage xanthomonadales_odb10) (12), which indicated that the CPBF 427 sequence is >99.8% complete. Default parameters were used for all software unless otherwise noted.

The CPBF 427 assembly has a total length of 5,228 Mb, comprising 1 circular contig with a G+C content of 65.38%. The annotation reported 4,367 coding sequences (CDSs) with 4,237 coding genes from a total of 4,465 genes, with 2 complete sets of rRNAs (5S, 16S, and 23S rRNAs), 54 tRNAs, 38 noncoding RNAs (ncRNAs), and 130 pseudogenes. Table 1 contains a summary of sequence data and genome statistics.

**TABLE 1:** Summary of sequence data and genome statistics

Parameter	Finding(s) for CPBF 427
ENA/GenBank accession no.	GCA_903989475
ONT sequencing	
No. of reads	14,302
Read length (mean ± SD) (nt)	6,358 ± 7,722
Read *N*_50_ (nt)	13,332
Illumina sequencing	
No. of reads	7,562,695
Read length (nt)	2 × 150
Mean coverage (×)	474
G+C content (%)	65.38
*N*_50_ (nt)	5,228,174
No. of sequences	1
Genome size (nt)	5,228,174
Total no. of genes	4,465
Total no. of CDSs	4,367
No. of coding genes	4,237
No. of CDSs with protein	4,237
No. of RNA genes	98
No. of rRNAs (5S, 16S, 23S)	2, 2, 2
No. of complete rRNAs (5S, 16S, 23S)	2, 2, 2
No. of tRNAs	54
No. of ncRNAs	38
No. of pseudogenes	130
No. of CDSs without protein	130

This single-scaffold genome may be a robust reference for comparative genomic studies of X. arboricola pv. juglandis, particularly important for addressing the genomic context of genetic determinants of pathogenicity and virulence. Furthermore, the CPBF 427 genome may contribute to the disclosing of putative overwinter adaptations of bacteria belonging to this taxon and to better understanding of its life cycle.

### Data availability.

The raw data and assembled/annotated sequence have been deposited in the European Nucleotide Archive (ENA). The study accession number is PRJEB39139, and raw Illumina and MinION reads are available under the accession numbers ERX2780812 and ERX4296810, respectively. The accession number for the assembled genome is GCA_903989475. Supplementary data and genome annotations are available via the BioStudies database accession number S-BSST427.
